# Projecting the impact of an ebola virus outbreak on endangered mountain gorillas

**DOI:** 10.1038/s41598-023-32432-8

**Published:** 2023-04-07

**Authors:** Dawn M. Zimmerman, Emily Hardgrove, Sara Sullivan, Stephanie Mitchell, Eddy Kambale, Julius Nziza, Benard Ssebide, Chantal Shalukoma, Mike Cranfield, Pranav S. Pandit, Sean P. Troth, Taylor Callicrate, Philip Miller, Kirsten Gilardi, Robert C. Lacy

**Affiliations:** 1 Veterinary Initiative for Endangered Wildlife, Bozeman, MT USA; 2 Smithsonian Institution, National Museum of Natural History, Washington, DC USA; 3 Department of Epidemiology of Microbial Disease, Yale School of Public Health, New Haven, CT USA; 4 Virginia-Maryland Regional College of Veterinary Medicine, Virginia Tech, Blacksburg, VA USA; 5 Species Conservation Toolkit Initiative, Chicago Zoological Society, Brookfield, IL USA; 6 Center for Species Survival, Smithsonian National Zoological Park and Conservation Biology Institute, Washington, DC USA; 7 Gorilla Doctors (MGVP, Inc.), Davis, CA USA; 8 Institut Congolais Pour La Conservation de Nature, Kinshasa, Democratic Republic of Congo; 9 EpiCenter for Disease Dynamics, One Health Institute, School of Veterinary Medicine, University of California Davis, Davis, CA USA; 10 Merck & Co., Inc., PA West Point, USA; 11 IUCN SSC Conservation Planning Specialist Group US, Apple Valley, MN USA; 12 School of Veterinary Medicine, Karen C. Drayer Wildlife Health Center, University of California, Davis, CA USA

**Keywords:** Conservation biology, Ecological epidemiology, Ecological modelling

## Abstract

Ebola virus is highly lethal for great apes. Estimated mortality rates up to 98% have reduced the global gorilla population by approximately one-third. As mountain gorillas (*Gorilla beringei beringei*) are endangered, with just over 1000 individuals remaining in the world, an outbreak could decimate the population. Simulation modeling was used to evaluate the potential impact of an Ebola virus outbreak on the mountain gorilla population of the Virunga Massif. Findings indicate that estimated contact rates among gorilla groups are high enough to allow rapid spread of Ebola, with less than 20% of the population projected to survive at 100 days post-infection of just one gorilla. Despite increasing survival with vaccination, no modeled vaccination strategy prevented widespread infection. However, the model projected that survival rates greater than 50% could be achieved by vaccinating at least half the habituated gorillas within 3 weeks of the first infectious individual.

## Introduction

The increasing threat of disease transmission among wildlife, domestic animals, and humans is predicated on the growth of human populations and subsequent land-use change, driving opportunities for disease spillover^1^. The world is enduring a tragic example in the COVID-19 pandemic, caused by a virus believed to have its origins in wildlife hunted or consumed by people. With ~ 72% of emerging infectious diseases (EIDs) originating in wildlife^2^, proactive measures at the human-wildlife interface are critical for the mitigation of EIDs. Less appreciated is the fact that these same drivers also lead to an increased risk of disease transmission from humans to wildlife. This is particularly evident in great apes, human’s closest relatives, which share susceptibility to many of the same pathogens causing illnesses in humans. There are numerous examples of wild chimpanzee and gorilla morbidity and mortality likely caused by infection with human pathogens^3–8^.

Ebola virus (EBOV) has caused significant mortality in both humans and great apes, estimated to have reduced the global gorilla population by approximately one-third^9^, with social impacts on gorillas likely persisting for years^10^, population recovery predicted to take decades^11,12^, and genetic impacts potentially persisting for centuries^13^. Western lowland gorilla population declines of 56–98% have been documented at study sites adjacent to human outbreak zones^14,15^. Seven EBOV outbreaks have been reported in great ape populations globally from 1992–2003 and Rizkalla et al.^16^ estimated a 63.6% chance of EBOV infecting a susceptible great ape population annually. Although this annual rate has decreased as there have been no outbreaks confirmed in great apes since 2005^17^, the virus is considered endemic in East-Central and West Africa, with concern for cross-species transmission in regions of intense human-wildlife overlap^18^.

**Figure 1:**
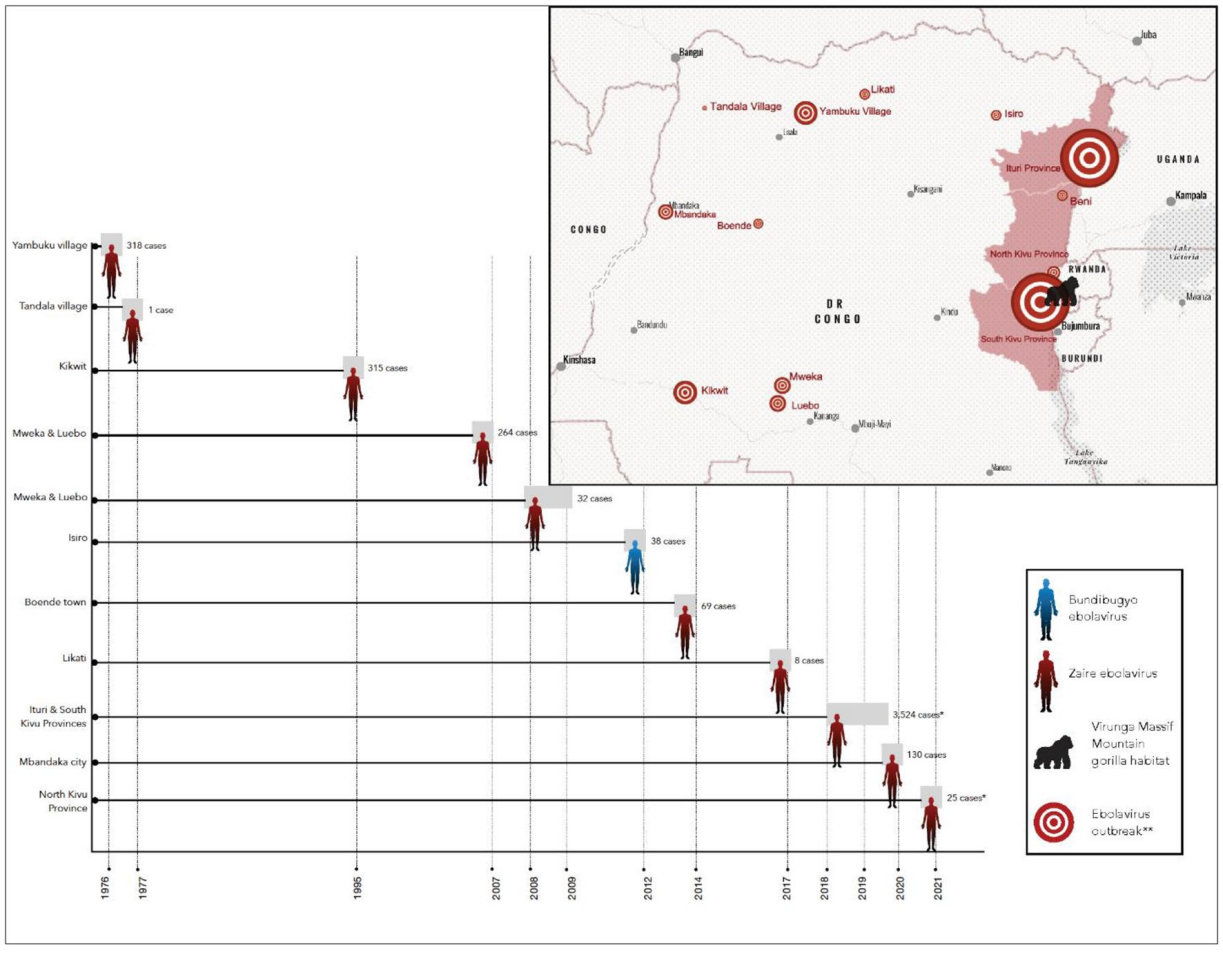
Timeline and locations of human Ebola virus outbreaks in the Democratic Republic of Congo (DR Congo). Outbreak data from: Centers for Disease Control and Prevention (2022). Map created with *ArcGIS Online* (Version 2.8), Esri Inc. https://www.esri.com/en-us/arcgis/products/arcgis-online/overview. *pooled from multiple outbreaks. **size of circle represents size of ebolavirus outbreak in local geographic area.

One such interface of concern is the Virunga Massif (VM) spanning the borders of Rwanda, Uganda, and the Democratic Republic of Congo (DRC). This landscape is home to one of only two populations of the endangered mountain gorilla (*Gorilla gorilla beringei*), supporting ~ 607 of the remaining 1063 individuals; the second population within Bwindi Impenetrable National Park, Uganda, is in proximity but isolated from the VM population^19^. While characterized as among the most biodiverse landscapes in all of Africa, the VM is threatened by illegal extraction of resources, civil unrest, wildlife poaching, increased numbers of livestock along the park borders, and pressure from surrounding communities that have some of the highest human population densities in continental Africa (up to 1000 people per square kilometer)^20^. This region has also experienced EBOV outbreaks (predominantly Zaire species), with 14 outbreaks occurring in DRC since the virus was first reported in the country in 1976^21^ (Fig. 1).

**Figure 2:**
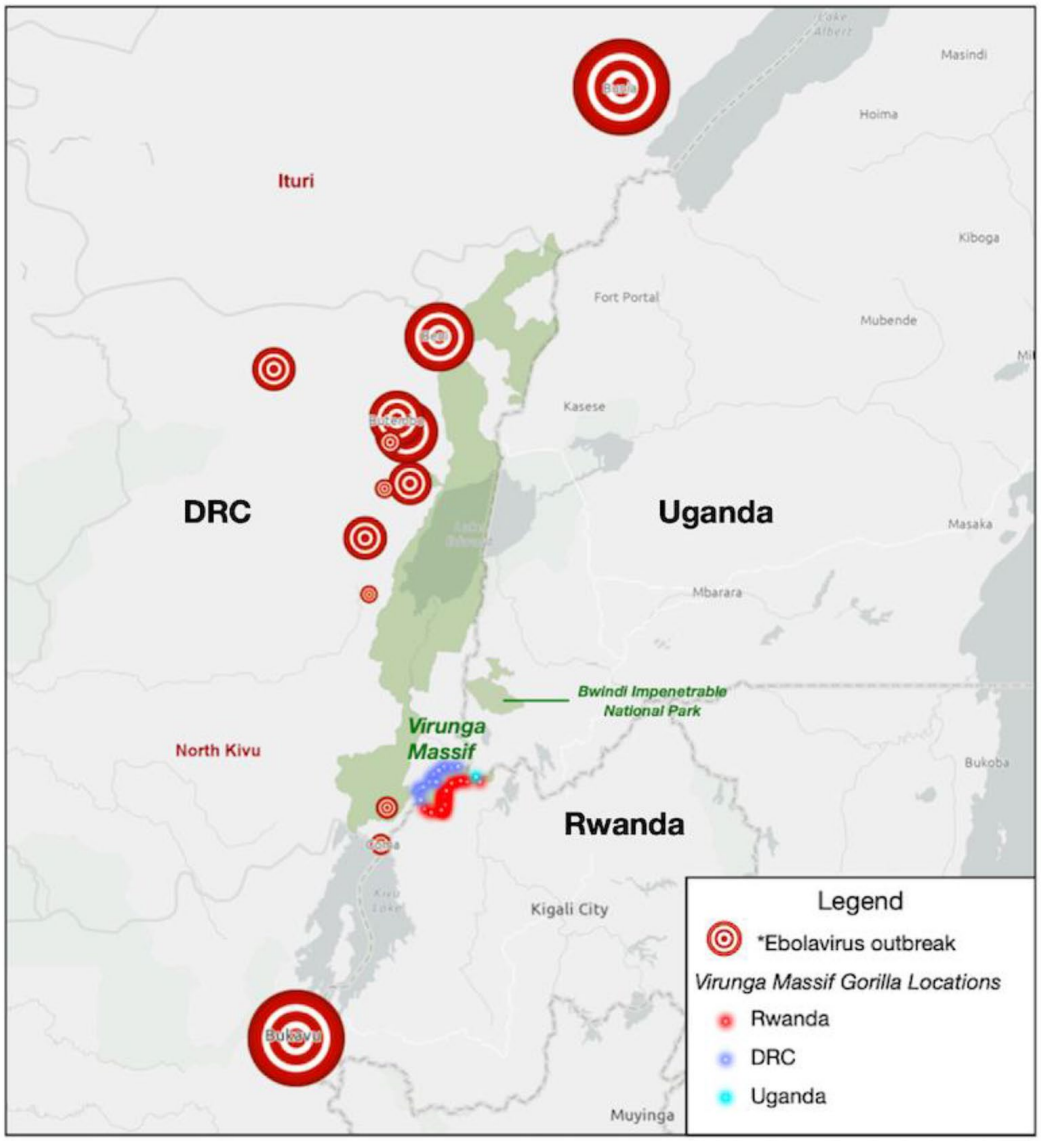
Locations of 2018–2021 human Ebola virus cases in the Democratic Republic of Congo (DRC) in proximity to the Virunga Massif and Bwindi mountain gorilla habitats. Outbreak data from: Centers for Disease Control and Prevention (2022). Map created with *ArcGIS Online* (Version 2.8), Esri Inc. https://www.esri.com/en-us/arcgis/products/arcgis-online/overview. *size of circle represents size of Ebola virus outbreak in local geographic area.

In 2018–2020, an EBOV outbreak (Zaire species) in eastern DRC—one of the six EBOV outbreaks in DRC since 2018^22^—came within approximately 80 km of the Mikeno sector of Virunga National Park^19^ (Fig. 2), posing a risk to the VM mountain gorilla subpopulation. It was the second largest EBOV outbreak in history with 2287 human deaths^23^. Control of transmission was hindered by sociopolitical gaps in public health response and insecurity of the region. Human presence in Virunga National Park, both legal (e.g. park personnel, tourists) and illegal (e.g. poachers, militia), created potential for introduction of EBOV to the park from an infected human. The risk of transmission to the VM mountain gorilla population was heightened with the potential for close proximity of gorillas and humans, as approximately 2/3 of the world’s mountain gorillas are human-habituated to facilitate ecotourism and research^19^.

While EBOV has not yet been detected in mountain gorillas, preparedness and response will be critical to survival of the subspecies. With the hypothesis that EBOV would rapidly spread among gorillas throughout this protected area and cause mass mortality, we used an individual-based simulation^24,25^ to predict the possible impact of Ebola Virus Disease (EVD) on the mountain gorilla population of the Virunga Massif. Then, we modeled the potential impact of EBOV vaccination of gorillas in reducing transmission and resultant losses.

## Results

### Modeling the spread of Ebola virus across mountain gorilla groups

**Figure 3:**
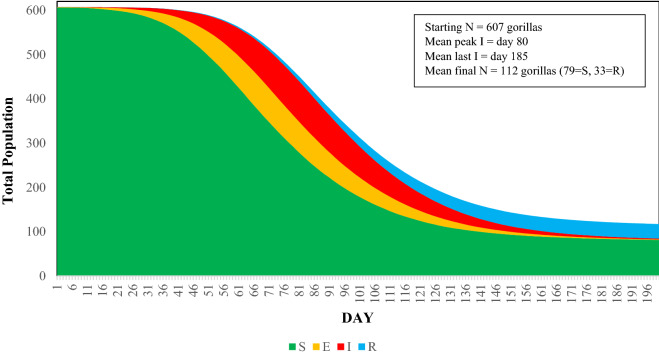
Proportions of SEIR states in the population and projected time course of Ebola virus transmission after entering the mountain gorilla population through one infected individual in the DRC-1 group at Day 0. S—Susceptible, never infected; E—Exposed and infected; I—Infectious; R—Recovered. Results averaged across 500 iterations.

Based on the model, contact rates among gorilla groups and lone silverbacks are high enough to allow rapid spread of EBOV through the Virunga Massif population, regardless of the scenario in which the first case occurs (Table 1). In the absence of protective vaccination, EBOV spreads rapidly among groups and lone silverbacks, eventually infecting 85 to 87% of the gorillas (Table 1). Only 6% of infected gorillas recover and survive. The epidemic peaks at approximately 10–14 weeks, and is resolved after 25–28 weeks (Fig. 3). The mean survival rate of those that escape infection or that recover from infection is 18–21% of the population, leaving 112–125 gorillas surviving out of an initial population of 607 (Fig. 3). In almost all (> 98%) iterations of each scenario, the epidemic caused the deaths of more than half of the gorillas in the Virunga Massif. The point of entry (location) of the first infected individual had little effect on the spread of infection throughout the population. Table 1 shows the mean and variation in results for five scenarios in which the initial infected gorilla was either an individual within one of four different groups or was a lone silverback.

**Table 1:** Summary impacts of a modeled Ebola virus outbreak entering the Virunga Massif population of mountain gorillas. Scenario = gorilla group of an initial infected individual in the Democratic Republic of Congo (DRC) or Rwanda (RW), or infected lone silverback (LSB); Cumulative Prevalence = proportion of the 607 gorillas that became infected; Duration = days to the disappearance of Ebola virus from the population; #S = final number of Susceptible gorillas, those that never become infected; #R = final number of Recovered gorillas (those that were infected but recovered); Survival Rate = proportion of the population that survived, as either Susceptible or Recovered. Presented as mean (SD); SD—standard deviation across 500 iterations. The SE of means would each be the SD/√500 = SD/22.36.

Scenario	Cumulative Prevalence	Duration	#S	#R	Survival Rate
DRC-1	0.869 (0.074)	185 (32)	79 (45)	33 (6)	0.185 (0.070)
DRC-2	0.871 (0.060)	179 (34)	78 (36)	33 (6)	0.184 (0.057)
DRC-3	0.871 (0.066)	183 (34)	79 (40)	33 (6)	0.184 (0.062)
DRC-LSB	0.855 (0.113)	181 (36)	88 (69)	33 (7)	0.199 (0.107)
RW-1	0.848 (0.151)	192 (39)	93 (91)	32 (8)	0.206 (0.142)

### Modeling the effect of vaccination to mitigate EVD mortalities

Prevalence of EBOV infection was assessed against 15 vaccination regimes (Table 2). None of the vaccination strategies tested were able to prevent Ebola from spreading throughout the majority of the population, resulting in the infection of at least 75% of the unvaccinated gorillas.

**Table 2:** Summary impacts of a modeled Ebola virus outbreak entering the Virunga Massif population of mountain gorillas via one exposed gorilla in the DRC-1 group, under various vaccination strategies. Preemptive = gorillas vaccinated prior to arrival of the first Ebola case; Week 3 = vaccinations administered 3 weeks after the first case becomes infectious; Week 6 = vaccinations administered 6 weeks after the first Infectious individual. # Gorillas Vaccinated was either applied randomly to a percent of the habituated gorillas or applied to 1 or 4 gorillas in each of the 33 habituated groups. #V is the number that were effectively vaccinated (resulting in protective immunity), i.e., not including the 3% for which the vaccine was not effective; other columns as in Table 1. Presented as mean (SD); SD = standard deviation across 500 iterations.

Vaccination scenario	#Gorillas vaccinated	Cumulative prevalence	Duration	#S	#R	#V	Survival rate
Preemptive	Percent						
	0	0.869 (0.074)	185 (32)	79 (45)	33 (6)	0	0.185 (0.070)
	10	0.789 (0.076)	189 (36)	76 (46)	30 (6)	52 (6)	0.261 (0.072)
	25	0.666 (0.083)	195 (38)	73 (50)	25 (6)	130 (10)	0.376 (0.078)
	50	0.437 (0.108)	201 (53)	83 (63)	17 (6)	259 (12)	0.591 (0.101)
	Per group						
	1	0.811 (0.072)	188 (36)	82 (44)	31 (6)	33 (1)	0.240 (0.068)
	4	0.633 (0.112)	189 (44)	93 (68)	24 (6)	130 (2)	0.408 (0.105)
Week 3	Percent						
	10	0.796 (0.059)	187 (32)	74 (35)	31(6)	50 (7)	0.254 (0.056)
	25	0.675 (0.063)	192 (35)	72 (37)	26 (5)	125 (10)	0.368 (0.060)
	50	0.471 (0.061)	197 (42)	70 (34)	18 (5)	251 (13)	0.558 (0.057)
	Per group						
	1	0.811 (0.081)	186 (36)	83 (49)	32 (6)	32 (1)	0.241 (0.077)
	4	0.654 (0.064)	185 (34)	86 (38)	25 (6)	124 (3)	0.388 (0.060)
Week 6	Percent						
	10	0.805 (0.059)	188 (33)	75 (35)	31 (6)	43 (8)	0.245 (0.057)
	25	0.708 (0.060)	190 (36)	70 (33)	27 (6)	108 (15)	0.337 (0.056)
	50	0.538 (0.062)	191 (39)	64 (26)	20 (5)	216 (25)	0.495 (0.058)
	Per group						
	1	0.817 (0.076)	182 (31)	84 (46)	31 (6)	27 (3)	0.235 (0.072)
	4	0.690 (0.066)	182 (34)	80 (39)	26 (6)	108 (9)	0.353 (0.062)

Vaccination scenarios yielding the highest survival (Table 2; Figure 6 ) included: preemptive vaccination of 50% of habituated gorillas (i.e., vaccinated prior to first infectious individual; mean survival 59%), vaccinating 50% of habituated gorillas at 3 weeks after the start of the outbreak (i.e., 3 weeks after first infectious individual; mean survival 56%), and vaccinating 50% of habituated gorillas at 6 weeks after the start of the outbreak (mean survival 50%). However, even in the best-case scenario in which infection enters the population with the vaccine having been administered to 50% of habituated gorillas (44% of the total population modeled; Table 3), 44% of the total population still becomes infected, and only 24% of the unvaccinated population (14% of the overall population) escape being exposed and remain Susceptible. If not enough individuals are vaccinated, or if vaccination occurs too slowly, cumulative prevalence can exceed 80%.

**Table 3:** Number of monitored and unmonitored Virunga Massif mountain gorillas in the DRC, Rwanda, and Uganda included in the *Outbreak* model, based on group sizes observed at the end of 2018.

	Monitored				Unmonitored				Virunga Massif total
	Groups	Individuals in groups	Lone individuals	Sub-total	Groups	Individuals in groups	Lone Individuals	Sub-total	
DRC	11	189	3	192	10	70	2	72	264
Rwanda	21	332	1	333	Not included	Not included	0	0	333
Uganda*	1	10	Not included	10	0	0	Not included	0	10
Virunga Massif Total	33	531	4	535	10	70	2	72	607

*Because only one group was located in Uganda, and was a group that sometimes came into Rwanda, we combined the Rwanda and Uganda groups into one dataset (RW-UG).

Delaying vaccination until 3 weeks after the first gorilla becomes Infectious results in only slightly more gorillas becoming infected and only slightly lower numbers of survivors of the epidemic in comparison to preemptive vaccination (Table 2). Despite the three-week delay, almost as many receive vaccine protection as in the preemptive scenarios because EBOV has not yet spread widely throughout the population. Delaying vaccination until 6 weeks after the first infectious individual results in higher disease prevalence, and decreases the overall survival rate.

### Preemptive (Preventative) Vaccination

**Figure 4:**
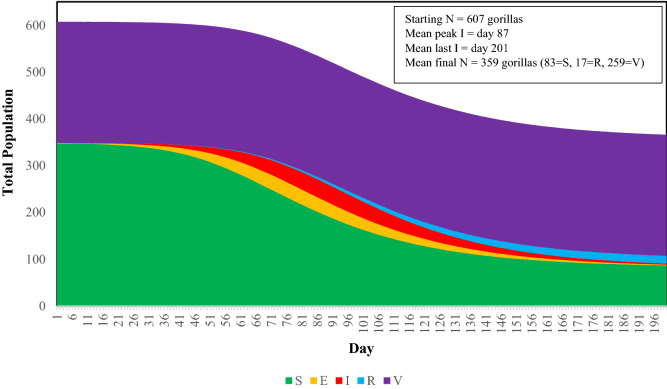
Proportions of SEIR states in the population and projected time course of Ebola virus transmission after entering the mountain gorilla population with one infected individual in the DRC-1 group at Day 0, when vaccinations had been administered preemptively to 50% of the habituated gorillas. S—Susceptible, never infected; E—Exposed and infected; I—Infectious; R—Recovered; V—Effectively vaccinated. Lines averaged across 500 iterations.

To achieve survival of more than half of the population using a preemptive vaccination approach, the model determined that at least 50% of the habituated gorillas would have to be vaccinated before EBOV spreads from the first infectious individual. Feasibly, this means that the gorillas would have to be vaccinated as a preventative measure, hence labeled as “preemptive”. This vaccination strategy resulted in a projected population survival rate of ~ 59% (359 of the initial 607 gorillas) (Table 2; Fig. 4).

#### Delayed Vaccination

**Figure 5:**
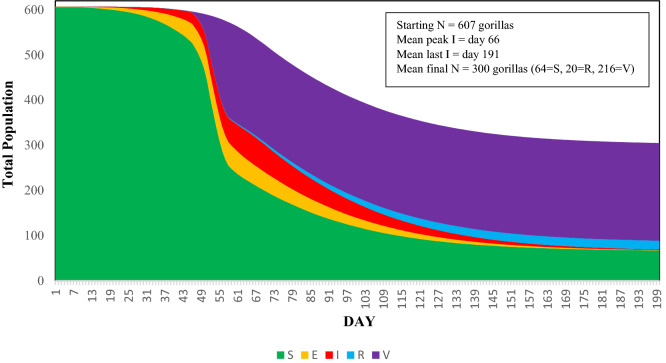
Proportions of SEIR states in the population and projected time course of Ebola virus transmission after entering the mountain gorilla population with one infected individual in the DRC-1 group at Day 0, when vaccinations are administered to 50% of the habituated gorillas 6 weeks after the first gorilla becomes infectious. S—Susceptible, never infected; E—Exposed and infected; I—Infectious; R—Recovered; V—effectively Vaccinated. Lines averaged across 500 iterations.

When protective vaccination does not occur until 3 weeks after the initial infectious gorilla, cumulative prevalence and therefore mortality is about 1% higher than in most comparable preemptive scenarios tested (Table 2). When vaccination does not occur until 6 weeks after the initial infectious individual, transmission among gorilla groups has already occurred and vaccination results in a mean of 9 or 59 more deaths than would have occurred if vaccination is preemptive, for strategies with 10% and 50% vaccination respectively (Fig. 5).

Vaccination results were very similar for all initial entry scenarios, with entry via DRC-LSB or RW-1 showing just slightly lower prevalence and slightly higher survival in scenarios with vaccination (data not shown), as was seen also in the scenarios without vaccination (Table 1).

### Sensitivity analysis

Sensitivity analyses showed (Figure S2, S3, S4, S5, S6) that the probability of encounters between groups (ENCFACTOR, applied as a proportion of the encounter rate assumed in the baseline scenario) had the largest effects of any of the tested variables (within the ranges examined), with higher encounter rates leading to more gorillas becoming infected (R^2^ = 0.78), a longer duration of the epidemic (R^2^ = 0.27), and fewer surviving (R^2^ =  − 0.83). Logistic regression of the probability of an epidemic infecting at least 50% of the gorillas, as a function of ENCFACTOR, showed that the encounter rate would need to be less than 37% of the baseline estimate to reduce the probability of an epidemic to less than 50% (Figure S7).

The model parameters of the incubation days (INCUBATION, time in the E state) and duration of infectivity (time in the I state, abbreviated as IDURATION) affected the time to the peak of the epidemic (R^2^ = 0.35 and R^2^ = 0.06, respectively) and the duration of the epidemic (R^2^ = 0.45 and R^2^ = 0.14). IDURATION also affected the number becoming infected (R^2^ = 0.36) and number surviving (R^2^ =  − 0.20), while INCUBATION had only weak effects on the number of gorillas infected (R^2^ = 0.03) or the number surviving the epidemic (R^2^ =  − 0.10) — the model outputs that summarize the severity of the epidemic. The recovery rate and the transmission rate, within the ranges tested, had relatively little effect on outcomes. Generalized Additive Model (GAM) fitted smooth curves for these impacts were consistent with the above trends, showing tight linear relationships for factors with strong correlations to outcomes and weak and often inconsistent trends for the weaker relationships (Figure S2, S3, S4, S5, S6).

Sensitivity analyses of the vaccination scenarios showed similar results to the no vaccination tests with respect to effects of disease parameters (i.e., encounter rate having a large effect on numbers of gorillas infected and surviving, incubation and infectivity duration affecting timing more than the severity of the epidemic, and recovery rate and transmission rate having relatively small effects on the timing and severity of the epidemic) (Figure S9, S10, S11, S12, S13). The effectiveness of the vaccine (VAXEFF, the proportion of the vaccinated individuals that acquire protective immunity) had relatively little effect on any of the output metrics. The percent of the population that was vaccinated (VAXPCT) had strong effects on the number infected (R^2^ =  − 0.36) and number surviving (R^2^ = 0.37), but had only weak effects on the time to the peak of the epidemic (R^2^ =  − 0.04) or the duration of the epidemic (R^2^ =  − 0.09).

## Discussion

This study modeled the effect of an EBOV outbreak on the endangered mountain gorilla population of the Virunga Massif, which spans protected areas in Rwanda, Uganda, and the Democratic Republic of Congo. We used the simulation model to ask the following questions: How fast will EBOV spread through the population? Will it move through the entire population, or burn out locally? How many gorillas will die? If it was possible to vaccinate, what regime would be optimal for protecting the largest portion of the population? The model projected that EBOV would spread through the population, from group to group, resulting in infection of 85–87% of the gorillas with 112–125 surviving out of an initial population of 607 individuals. While vaccination improved survivability, none of the vaccination strategies inhibited the spread of Ebola through much of the population and at least 75% of unvaccinated gorillas were therefore still infected. In order to increase survival to 59% (359 of the 607 individuals), the model showed that 50% of the habituated gorillas (~ 44% of gorillas across the Virunga Massif) would need to be preemptively vaccinated. That said, we found that when vaccination does not occur until 3 weeks *after* the initial gorilla becomes infectious (on average 8–10 days after infection), survivability is almost as high as with preemptive vaccination (~ 56%). This is likely because protective immunity from the vaccination is achieved before EBOV has spread far beyond the initial group. We therefore project that vaccination prior to the outbreak up until 3 weeks after the first infectious individual results in greater than 50% survival. It should be noted, however, that the loss of nearly 50% of the population, while it is certainly better than the loss of more than 80% as projected when no gorillas are vaccinated, would still be a devastating impact on the mountain gorilla population, with most unvaccinated gorillas succumbing to the disease.

It is also important to note that the delay to immunity from vaccination in the model encompasses the time to detect EBOV infection (mean duration of infectiousness is 12 days + / − 2 days)^26–28^, the time to administer the vaccine, and the time for the vaccine to become protective against infection. Therefore, achieving only a 3-week delay with a reactive vaccination strategy would require vigilant monitoring for disease and rapid response by field teams to administer vaccinations, especially considering that first detection would happen after the gorilla has become infectious for others. Furthermore, vaccinating 50% of the habituated gorillas would be a massive effort that could take significant time—likely more than 3 weeks even if an EBOV-infected individual is identified immediately after infection. Therefore, vaccination as a feasible mitigation strategy would ideally be performed preemptively, prior to the first individual becoming infectious.

Predictive models such as the one presented here can be instrumental in preparing and responding to a disease outbreak in a fragile wildlife population and are therefore important for making informed wildlife conservation management decisions^29,30^. Decision-making on mitigation efforts is then science-based, and facilitates cross-sectoral collaboration on data collection, sharing, and analysis, because the development of accurate models requires detailed data on a species, ecosystem, and epidemiological level^31^. This study used *Outbreak* software, an open-source tool that allows wildlife conservationists who may or may not have an extensive foundation in mathematical modeling, to predict impacts of disease in a population or ecosystem. While disease modeling of wildlife has unique challenges compared to human or livestock populations, due to limited data, conducting such exercises can contribute to our understanding of disease risk, make quantitative predictions for the future course of an outbreak, and inform population management strategies and interventions such as the effect of various (e.g. vaccination) scenarios before integrating into infectious disease risk protocols^29,30,32–35^. This is especially important for diseases with high mortality rates or those zoonotic in nature.

Although some wildlife population models must rely on theoretical data to inform input parameter values, mountain gorillas are unique in that they are closely monitored and individually recognizable (if habituated), allowing a valuable opportunity to include accurate home ranges, social networks, and movement data into the model. Contributing to the transmission dynamics of the model, actual GPS coordinates and recorded interactions among groups informed data-driven suppositions from veterinarians and scientists who track this population regularly. As this level of detail is not available for many wildlife populations, sensitivity testing of the model parameters is used to demonstrate what data at a population level most influences the model outcome and therefore what data should be integrated into routine population surveillance. The encounter rate between individuals is one input variable that can be very difficult to measure without the almost daily location data that have been collected for mountain gorilla groups. Even with daily records of group locations, we had to rely upon expert opinion to translate the observed inter-group distances into likelihood of direct or indirect contact (as via substrates) that could result in transmission of Ebola. Sensitivity tests showed that the effective encounter rate has a larger effect on the severity and time course of the epidemic than did the disease parameters that are often easier to measure and more narrowly estimated, e.g. transmission probability, incubation time, duration of infectivity of diseased individuals, and recovery rate. However, ominously for the gorillas, the encounter rate would have to be less than 37% of our baseline estimate for an outbreak of Ebola virus to not spread widely through the Virunga population. Quantifying the significance of these parameters is an important consideration for prioritizing data collection when planning monitoring programs and research studies, particularly of less-monitored species. This necessitates understanding the stochasticity and resilience of a system which models can help answer.

To the authors’ knowledge, this study is the first to model use of a human vaccine to stop the spread of a highly fatal zoonotic disease in an endangered wildlife population. Due to their genetic similarity, great apes and humans are not only susceptible to many of the same pathogens^36–38^ but also share many epidemiological parameters allowing for extrapolation across species models^39^. Broadly, better understanding disease dynamics in wildlife can not only inform the risk posed to adjacent human communities^1^, but also to the survival of endangered species. This duality is important to address, because with the increasing overlap of human-animal populations worldwide, the risk of disease transmission^40^ between humans and great apes heightens. With this recognition, we can start to put tools in place—such as outbreak modeling-to be more proactive than reactive towards disease risks.

All disease models come with limitations, and certainly cannot be validated unless an outbreak occurs as surmised in the model. Importantly, given the sporadic nature of EBOV, uncertainty surrounding the reservoir, and rapid spread^41,42^ upon entry regardless of source (as predicted by the model), this model did not investigate how Ebola virus would be introduced to the Virunga Massif gorilla population. Further investigation into Ebola virus transmission dynamics could help inform this model but was outside the scope of this project. In addition, as we were not modeling the impact over multiple years, we did not require estimates of the likelihood of EBOV entering the population. We instead focused on the consequences of even a single mountain gorilla becoming infected. To expand the model to include multiple years of potential outbreaks, we would need to model repeated infections over a number of years. We would also need to model the risk of infections from outside sources. This would be difficult given that the wildlife source of Ebola virus is still not definitively known^43^, nor is the prevalence in, and likelihood of, transmission from other non-human primates. We instead focused on mitigating EBOV spread once in the population, in order to better prepare and respond if an outbreak were to occur.

Another limitation was that the contact between groups was estimated at the group level, not on an individual level. As such, the model is not representative of an individual that may stray quite far from the group’s central location over any given day. If this individual is a source of infection or acquires infection when apart from the group, our model underestimates transmission of the virus. As our model already calculated an estimated high (80%) mortality rate without vaccination, we did not explore this complex possibility. Also of note is that the model did not include unhabituated gorillas in Rwanda, estimated at just 10 individuals as of the 2015–2016 census^19^, which would have provided additional routes for increased transmission among groups while increasing the number of gorillas that are unable to be vaccinated–overall, increasing spread and mortality of an EBOV outbreak. Moreover, as there are ongoing studies on the duration of protective immunity of the vaccine (S.P. Troth, personal communication), the vaccine parameters may have to be adjusted if protection is found to wane before the 12-month timeline examined in this model. Further study should also explore other possible vaccination strategies (e.g., ring vaccination based on location of initial infection) and a risk–benefit analysis.

Another goal is to complement this disease risk and management model with a population viability analysis on the modeled population “remnant” using *Vortex* software^44^ to predict the long-term impacts of EBOV on population structure and, hence, recovery. *Vortex* has previously been utilized for population viability assessments in many endangered species worldwide, including western lowland gorillas as well as mountain gorillas in the late 1990’s^45^, and has been used in conjunction with *Outbreak* to evaluate the impacts of infectious disease on population viability^29,35^. For this model, *Vortex* could be integrated to compare the long-term viability of the remnant population, with or without vaccination, after the EBOV outbreak ends. Future EBOV models should thus include the mountain gorilla subpopulation in Bwindi Impenetrable National Park in Uganda for a holistic prediction for the sub-species as a whole.

In addition to further quantifying the long-term impacts of vaccination, the combination of *Vortex* and *Outbreak* in such a well-studied species as the mountain gorilla will help in the continued refinement of the software and serves as a demonstration of disease modeling needs, capabilities, and contribution to short and long-term wildlife conservation goals. As a model is only as accurate as its data allows, this study also serves to identify gaps in data collection that must be filled in order to build an efficient model, as well as guide the future research focus of conservationists in providing foundational parameters needed for disease outbreak modeling in other species.

## Methods

### Modeling the spread of EBOV across mountain gorilla groups

The *Outbreak* model of infectious disease (version 2.14, open access software^24^ and manual^25^ was used to explore the possible spread of EBOV in the Virunga Massif mountain gorilla population if the virus entered the population via a single infected gorilla. *Outbreak* is an individual-based SEIR simulation that tracks the transition of individuals among Susceptible, Exposed (infected), Infectious, Recovered and resistant, and optionally Vaccinated states, according to specified contact rates with infected individuals or external disease sources, transition probabilities, and durations of states. We simulated encounters, disease transmission, and transitions among disease states of individuals on a daily basis for one year. *Outbreak* models all transition stochastically, occurring for each individual as simulated Bernoulli processes with the specified probabilities. Therefore, by running the simulation many times (we used 500 iterations for each of the scenarios), the model generates the mean and distribution of projected population outcomes. *Outbreak* provides for any probabilities or durations to be functions of population or individual characteristics^30^, importantly including spatial location and proximity to other individuals – thereby allowing for detailed modeling of the transmission of disease in populations with social structure and group cohesion. *Outbreak* includes a basic demographic model of births and non-disease related deaths^25^, although for modeling the spread of EBOV in mountain gorillas we did not invoke the demographic component (other than disease-caused deaths), because the rapid spread of the virus – with the disease running its course usually within 200 days – meant that the few births and natural deaths in that time would not have major impact on the dynamics of disease transmission.

#### Gorilla Population Size and Structure

Sociodemographic data on habituated gorilla groups and lone silverbacks in the Virunga Massif were obtained from the national wildlife authorities of Uganda (Uganda Wildlife Authority), Rwanda (Rwanda Development Board), and Democratic Republic of Congo (Institut Congolais pour la Conservation de la Nature). Additionally, approximate location and size of unhabituated groups and lone silverbacks were also obtained as available from population surveys. Although the number, composition, and location of groups are dynamic through time, we hypothesized that the exact number and configuration of groups would not have a major effect on the spread of EBOV through the population from one infected gorilla, nor would it be substantially affected by location of the first infected gorilla—as demonstrated in comparisons among alternative scenarios (see Table 1).

Habituated mountain gorillas (representing ~ 70% of the Virunga Massif population; Hickey 2019^19^) are well-monitored. We were therefore able to obtain data on each habituated gorilla group in all three countries based on monitoring records (to include the number of gorillas by sex and age class), known lone silverbacks (solitary males), and 10 known but unhabituated groups in the DRC (Table 3), for a total of 607 gorillas used in the model. Because only one group was located in Uganda, and was a group that sometimes came into Rwanda, we combined the Rwanda (RW) and Uganda (UG) groups into one dataset (RW-UG).

### Model Scenario: EBOV Entry

We modeled several possible points of entry of EBOV into the Virunga Massif mountain gorilla population through five scenarios: 1) infection of a lone silverback ranging along the park boundary in the DRC (DRC-LSB), 2–4) infection of a single individual in groups of varying sizes (n = 10, 19, and 30 gorillas) in DRC (DRC-1, DRC-2, DRC-3), and 5) infection of a single individual in a large group (n = 24) in Rwanda (RW-1) (Table 1; Figure S1). The results showed that the spread of EBOV was insensitive to where the infection first entered the population, so further testing of possible entry points was not warranted. We also did not compare the impact of age class or sex of the first infected individual, as we have no data to indicate that transmission dynamics differ among age and sex classes. We therefore assumed Ebola virus transmission dynamics were consistent across gorilla demographics.

#### Contact rates and disease transmission probabilities

Not only is there evidence that Ebola virus can rapidly transmit within a gorilla group^46^, there have also been reports of intergroup transmission. Walsh et al.^26^ reported observations of western lowland gorillas inspecting dead or dying gorillas from other groups as well as feeding from the same trees as other groups in close succession, increasing the risk of infection spreading by tissue, blood, feces, urine, or saliva of non-group individuals. Given this risk of transmission and applying it to the different group dynamics of mountain gorillas, we modeled disease transmission of mountain gorillas as occurring between overlapping groups using geospatial (GPS) data from daily tracking records.

Once EBOV was introduced into the model population, we determined the likely spread and severity, modeling projected daily disease dynamics for a subsequent one-year period. The rate of contact between mountain gorilla groups was modeled using the recorded geographic locations of habituated groups and social dynamics of the Virunga Massif mountain gorilla population. The extensive monitoring of habituated gorillas enabled group composition and GPS locations to be captured almost daily, and often multiple times in a day. GPS data were available for groups in DRC from 01 January 2014 to 24 March 2019, with a mean of 1090 (median 471) sightings per group. GPS data were available for groups in Rwanda from 01 January 2018 to 31 December 2018, with a mean of 723 (median 413) sightings per group. Mean locations of each group are shown in Figure S1. Local experts provided estimates of the probability of an effective “contact” between two groups based on their proximity on any given day (Table S1). We defined such “contact” as two distinct groups or lone males using nearby or overlapping habitat locations on the same day, as EBOV transmission could occur either directly between gorillas (including contact with carcasses of dead animals) or indirectly through contact with a common substrate. We do not have quantitative data on the effective contact rate as a function of distance between any pair of groups, so we relied on expert opinion from field biologists (Table S1). Our sensitivity tests examined a full range of encounter rates (Table 4) to explore the impact of this uncertainty in encounters capable of causing transmission as a function of mean inter-group distance.

**Table 4:** Model parameters varied in sensitivity analyses of effects on the severity and timing of a simulated Ebola virus outbreak. All parameters were varied independently and simultaneously in 10,000 iterations of one scenario that did not include vaccination (results for the first five disease parameters) and of one scenario that included preemptive vaccination of 50% of the gorillas in habituated groups (results shown for the last two vaccination parameters). R^2^ was calculated for the duration of the epidemic (Epiduration), total number of individuals alive at the end of the simulation (N), the number of highest cases in a single day (peak IE), and the day with the highest number of cases (peak day).

Model parameters	Description of the parameter	Values tested	Sensitivity results (Epi duration, N, PEAKIE, Peak Day)
ENCFACTOR	Multiplier factor applied to encounter rates between groups	Sampled from Uniform [0, 1]) (base: 1)	(0.27, − 0.83, 0.78, 0.01)
TRANS	Transmission rate, per encounter	sampled from Uniform [0.05, 0.10] (base: 0.067)	(− 0.09, − 0.05, 0.1, − .0.09)
INCUBATION	Incubation (duration of E, in days)	sampled from integer Uniform [5, − 15] (base: 10, with SD = 3)	(0.45, − 0.1, 0.03, 0.35)
IDURATION	Duration of disease (in days)	sampled from integer Uniform [6, −18] (base: 12, with SD = 2)	(0.14, − 0.2, 0.36, 0.06)
RECOVERY	Recovery rate	sampled from Uniform [0.02, 0.12] (base: 0.063)	(− 0.02, 0.06, − 0.01, − 0.01)
VAXPCT*	Percent of habituated population vaccinated	sampled from CHOOSE(10;25;50)	(− 0.09, 0.37, − 0.36, − 0.04)
VAXEFF*	Efficacy of vaccine as probability of immunity	sampled from Uniform [0.9, 1.0]; (base: 0.97)	(0.0, 0.02, − 0.03, 0.0)

*Only used in vaccination scenarios.

For each day when GPS coordinates were available for both of a given pair of groups or lone males, we first found the average distance between the two groups for that day (using geodesic distance which is appropriate for GPS coordinates). As explained further below, we then estimated frequency of encounters between the groups based on estimates of contact rates averaged across days in which locations of both groups were recorded.

We used these probabilities along with the average daily distance between each pair of groups to calculate the daily probability of contact between the pair. From the full set of daily distances (re-scaled to units of 250 m to fit a 140 × 100 grid for convenience of modeling) and encounter probabilities between each pair of groups, we calculated the average daily encounter rate on days when locations of both were recorded. We could not directly use these observed between-group encounter rates to specify the probability of contact for all pairs of groups, because for some pairs of groups there were no days on which both were observed, or so few days that the data would not provide reliable estimates of encounter rates. Moreover, only approximate locations (and not daily sightings) were available for unhabituated groups. We therefore used the data we had for pairs of groups that had 10 or more days in which the locations of both were recorded to calculate via regression analysis the relationship between mean encounter rate (as calculated from the distances between them on days in which both groups were observed) and the distance between mean locations (averaged over all days in which a group was seen). We found that the relationship between encounter rate and mean distance that best fit the data was proportional to the inverse square-root of distance, i.e., Encounter Rate = B/(Distance^2^). We did not include an intercept in the regression, because as Distance becomes large, the Encounter Rate will necessarily approach 0. We calculated the relationship both separately for DRC and RW-UG groups, and with both sets of data combined. We had only sparse GPS data (fewer than 10 recorded points) on locations of 3 lone males in DRC, so we used those data to estimate mean locations, but not for calculating the encounter-distance functional relationship. For one lone male in Rwanda, we had GPS data on 65 daily locations, and we included those data in the estimation of the encounter-distance relationship. Although the data on encounters between this lone male and groups did not suggest a different frequency of encounters than between pairs of groups of similar distance, we cannot evaluate if the contact and transmission rates between lone males might be different than between groups. Lone males or group members that stray from their group for a period of time could create a route for greater spread of Ebola.

For the DRC habituated groups, the relationship between encounter rate and mean distance fit the inverse distance-squared regression with B = 4.7028 (on the re-scaled 250 m grid coordinates; B = 0.2939 when scaled to distance in km), with the variation of individual pairwise encounters around the regression line being given by SD = 0.0201. The data fit the line well, with R^2^ = 0.774, while other regressions, such as an inverse function, other exponential functions, log functions, or weighted regressions, did not fit the data as well. For the RW-UG groups the relationship between encounter rate and mean distance fit the inverse distance-squared regression with B = 0.6224, with the variation of individual pairwise encounters around the regression line being given by SD = 0.0067, and R^2^ = 0.707. For the combined DRC + RW-UG data, the relationship between encounter rate and mean distance fit the inverse distance-squared regression with B = 0.8032, with the variation of individual pairwise encounters around the regression line being given by SD = 0.0167, and R^2^ = 0.360.

Estimated encounter rates between groups in RW-UG were lower than between groups in DRC with similar mean distances, because GPS location data showed that pairs of groups in RW-UG come into near proximity less often than do pairs of groups in DRC that have similar distances between mean locations. Consequently, although the RW-UG families are clustered more tightly within a smaller overall range than are DRC families (see Figure S1), the estimated between-group encounter rates are quite similar. For encounters across the border, we used the regression that was based on both sets of data (DRC and RW-UG). This functionally models the encounter rates across the border as being intermediate between what is observed in DRC and what is observed in RW-UG (but more like the RW-UG regression).

We also needed to model unhabituated DRC groups for which we had no daily GPS data. Based on a simple map of approximate locations of these unhabituated groups during the 2015–2016 survey^19^, we semi-randomly assigned a location for each group that was a little to the south of a randomly selected habituated group. The locations for the unhabituated groups were re-assigned with each iteration of the simulation of the spread of EBOV, so as to project the disease dynamics based on a plausible sampling of locations for those groups. The encounter rates for unhabituated groups to each other group were then estimated by the regressions described above.

In this way, each pair of groups in our model (including both habituated and unhabituated) was assigned an encounter rate based on the distance between the groups. Encounters of individuals of one group to individuals in a neighboring group will not be independent, because groups move through the home range as a largely cohesive group. Therefore, when contacts between groups were determined as outcomes of the Bernoulli process with the probabilities specified from the regressions described above, the result (contact or not on any given day) was forced to be the same for all individuals within each group. This might overestimate the cohesiveness of groups. However, if group members disperse far enough apart so that some have less synchronous contacts with neighboring groups, a consequence would be that our model would underestimate the rate at which EBOV would be transmitted between groups. There are additional unhabituated groups and lone males in DRC and Rwanda for which we have no data on location, and those unmonitored gorillas were not included in our model. These unidentified animals might provide another route for disease transmission between groups, and this would be another cause of underestimation of the spread and population impact of Ebola.

After EBOV enters a group, the contact rate between individuals within that group was estimated by field biologists to be 0.95/day, thereby virtually assuring that the disease would spread throughout a group once it entered. However, “contact” between an infected gorilla and others, as described above, would only sometimes result in transmission of the virus. The true transmission probability for gorillas is unknown, but Rizkalla et al*.*^16^ estimated a rate of 0.5 over the 10 days that an animal would typically be infectious. We therefore set the probability of transmission between an infectious gorilla and a susceptible gorilla over a single day during which an encounter between them occurred to be 0.067 in our initial models, and we tested values ranging from 0.05 to 0.10 in sensitivity analyses.

#### Virus Characteristics and Infection Outcomes

Estimates of how EBOV would likely affect gorillas that become infected was extrapolated from data on the disease in humans and non-human primates, including from previous EBOV-Zaire outbreaks in western lowland gorillas^16^. All values for epidemiological characteristics (e.g., transmission dynamics, infectivity, incubation time, virulence, length of disease, mortality rates) used in our model and the ranges of values tested in sensitivity analyses are listed in Table S1. Brief explanations of some of the key disease parameters are described here. We set the duration of infectiousness to be sampled from a normal distribution with mean 12 days and SD = 2 days, based on the reports of about 8 days to death for humans that are not treated^27^ and an assumption that carcasses of gorillas that died from EBOV would remain infectious for about 4 days after death^28^. We used the Rizkalla et al.^16^ estimate of 0.063 for the survival rate for infected gorillas, and we assumed that recovered individuals would be resistant to reinfection for at least a year, and therefore through the duration of the local outbreak (in that, the disease was shown to run its course over 200 days). Cumulative prevalence was calculated by summing new infections over the entire model year and dividing by the initial population size.

### Modeling the effect of vaccination to mitigate EVD mortalities

**Figure 6:**
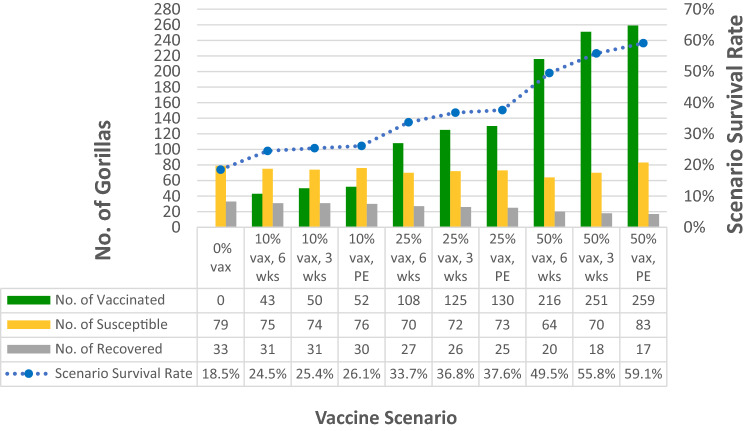
Mean end counts (the number of surviving individuals at the end of the year) by disease state listed in order of least survival to greatest survival for the various vaccination scenarios. This demonstrates that the pathogen sweeps through the population in every scenario. PE—preemptive; vax—vaccination of habituated gorillas.

With the *Outbreak* model, we also examined how various possible vaccination strategies would change the likelihood of widespread infection (an “outbreak” or epidemic) and the number of gorillas that would survive an outbreak. As the vaccine we modeled was originally tested in non-human primates^47^, we expect it to be effective in mountain gorillas. However, the logistics of vaccinating a wild population, even a habituated one, would be challenging compared to lab conditions. In addition to difficulties in maintaining a cold chain until administration, there could be incomplete dose delivery when vaccinating via remote (dart) injection as well as variability in location of injection which could alter the absorption of the vaccine. In order to evaluate whether such an effort would be warranted, we assessed the impact of multiple scenarios for preemptive vaccination (defined as vaccination prior to the first infectious individual) and delayed vaccination (modeled at both 3 weeks and 6 weeks after the first individual becomes infectious) (Table 2, Fig. 6). These time frames were selected based on expected response times from the veterinary team. Three weeks was considered the shortest amount of time it would take to detect the outbreak, implement the vaccination strategy, and achieve immunity from vaccination. In addition, we modeled the effect of vaccination based on the number of gorillas vaccinated, which included a percentage of the habituated gorillas (10%, 25%, and 50%), randomly distributed across all gorillas in habituated groups, as well as a set number of individuals independent of the number of gorillas in the group (1 individual and 4 individuals per habituated group) (Table 2; Fig. 6). The scenarios testing a fixed number of vaccinations per group were examined because it is likely that one gorilla per group could be vaccinated, and not very feasible that more than four gorillas in a group could be vaccinated (on a given day) before the entire group would flee from the vaccination team. For the maximum number of animals vaccinated per group, the vaccination team would need to include multiple team members darting at once as well as multiple days if possible.

The vaccine used in the model was the rVSVΔG-ZEBOV-GP (V920), a live-attenuated, replication-competent, single-dose vaccine (Merck and Co, Inc, Rahway NJ). Although efficacy studies are still ongoing, initial results reported by the World Health Organization found this vaccine provided 97.5% protection from symptoms^47^. Duration trials are also still in progress. So far, no infections followed an 84-days vaccine trial published in 2017^48^. For the purposes of this model, we assumed protection would be conferred for the entire year of the model. We also assumed the vaccination would have not been protective or have reduced the severity of disease if administered after a gorilla was already infected with EBOV. The time between vaccination administration and protection was not included in the model; however, studies indicate that a single IM injection of V920 provided partial protection against lethal IM challenge (ZEBOV-Makona strain) 3 days post-vaccination and complete protection against challenge 7 days postvaccination^49^. Vaccine parameters used in the model are included in Table S1.

### Sensitivity analysis

The results of any model are dependent on the accuracy of the specified disease parameters, some of which may be estimated or extrapolated with varying confidence. In order to test how one or a group of parameters may affect the model’s results, sensitivity analysis is used. In this study, we tested a plausible range of values for each of the key disease parameters in the model, by sampling from those ranges in 10,000 repeats of the simulation. The sensitivity of model parameters was assessed as the total number of individuals alive at the end of the simulation (N), the day with the highest number of cases (peak day), the number of highest cases in a single day (peak IE), and the duration of the epidemic as model outputs. Specifically, sensitivity scenarios were simulated for DRC-1 as an entry point for the infection (i.e., the model initiation with the first case in the DRC-1 group on the park boundary) with and without preemptive vaccination. Model parameters and their values tested sensitivity are given in Table 4. For each outcome of interest from model simulations (N, peak day, peak IE and epidemic duration), a two-stage analytical approach was implemented. First Pearson’s correlation coefficient was calculated to understand effect size. Following that, multivariable generalized additive models (GAMs) were fitted with model parameters as covariates and simulation outcomes as dependent variables. Partial dependence trend lines of fitted smooths were plotted to understand the association between model parameters and simulation outcomes.

## Supplementary Information

Supplementary Information.

## Data Availability

Part of the datasets generated and/or analyzed during the current study are not publicly available due to security concerns in revealing precise locations of gorilla groups, but are available from the corresponding author on reasonable request.
