# Supplementary material for: Projecting the impact of an ebola virus outbreak on endangered mountain gorillas
Source: Sci Rep. 2023 Apr 7;13:5675. doi: 10.1038/s41598-023-32432-8 (PMC10082040; doi:10.1038/s41598-023-32432-8)
Supplement: Supplementary file 1 — Supplementary Information. [file 41598_2023_32432_MOESM1_ESM.docx]

**SUPPLEMENTAL**

**Projecting the Impact of an Ebola Virus Outbreak on Endangered Mountain Gorillas**

Dawn M. Zimmerman, DVM, MS,^1,2,3*ϯ^ Emily Hardgrove, DVM, MPH,^4ϯ^ Sara Sullivan, MS,^5^ Stephanie Mitchell, MS, RD, MPH,^6^ Eddy Kambale, DVM, MSc^7^ Julius Nziza, DVM,^7^ Benard Ssebide, DVM,^7^ Chantal Shalukoma, PhD, ^8^ Mike Cranfield, DVM,^7^ Pranav Pandit,^9^ Sean P. Troth DVM, PhD, Dipl ACVP,^10^ Taylor Callicrate, PhD,^5^ Philip Miller, PhD,^11^ Kirsten Gilardi, DVM, Dipl ACZM,^7,12ϯϯ^ and Robert C. Lacy, PhD^5ϯϯ^

* Corresponding author; dawn.zimmerman@viewildlife.org

^ϯ^Joint first authors

^ϯϯ^Joint senior authors

^1^Veterinary Initiative for Endangered Wildlife, Bozeman, MT, USA

^2^Smithsonian Institution, National Museum of Natural History, Washington DC, USA

^3^Department of Epidemiology of Microbial Disease, Yale School of Public Health, New Haven, CT, USA

^4^Virginia-Maryland Regional College of Veterinary Medicine, Virginia Tech, Blacksburg, VA, USA

^5^Species Conservation Toolkit Initiative, Chicago Zoological Society, Brookfield, IL, USA

^6^ Center for Species Survival, Smithsonian National Zoological Park and Conservation Biology Institute, Washington DC USA

^7^Gorilla Doctors (MGVP, Inc.), Davis, CA, USA

^8^Institut Congolais pour la Conservation de Nature, Kinshasa, Democratic Republic of Congo

^9^ Epicenter for Disease Dynamics, One Health Institute, School of Veterinary Medicine, University of California Davis, Davis, CA, USA

^10^Merck & Co., Inc., Sumneytown Pike, West Point, PA, USA

^11^IUCN SSC Conservation Planning Specialist Group US, Apple Valley, MN, USA

^12^Karen C. Drayer Wildlife Health Center, School of Veterinary Medicine, University of California, Davis, CA, USA

Figure S1. Illustrative maps to depict mean locations of habituated mountain gorilla groups in the Virunga Massif, with overlap zones of 500 (a), 1000 (b), and 2000 (c) meters around the center of the observed locations for each group. Note that even when two groups have mean locations > 1000m apart, on some days they can be observed to be in close proximity (e.g., < 100m apart) and therefore capable of transmitting EBOV. Scenarios of entry points of EBOV displayed on map: 1 = DRC-1; 2= DRC-2; 3 = DRC-3; LSB = DRC-LSB, infection of a lone silverback ranging along the park boundary in the DRC; and RW-1 = infection of a single individual in a large group in Rwanda. Map created with *ArcGIS Online* (Version 2.8), Esri Inc. [https://www.esri.com/en-us/arcgis/products/arcgis-online/overview](https://nam02.safelinks.protection.outlook.com/?url=https%3A%2F%2Fwww.esri.com%2Fen-us%2Farcgis%2Fproducts%2Farcgis-online%2Foverview&data=05%7C01%7CZimmermanD%40si.edu%7Cd2c733afda694ac1644c08db15a6d287%7C989b5e2a14e44efe93b78cdd5fc5d11c%7C0%7C0%7C638127578489213119%7CUnknown%7CTWFpbGZsb3d8eyJWIjoiMC4wLjAwMDAiLCJQIjoiV2luMzIiLCJBTiI6Ik1haWwiLCJXVCI6Mn0%3D%7C3000%7C%7C%7C&sdata=ro7qjQx8FAMJGx1YXetSO%2Bxo3VEAzxeCt7m0AzFfvCg%3D&reserved=0).


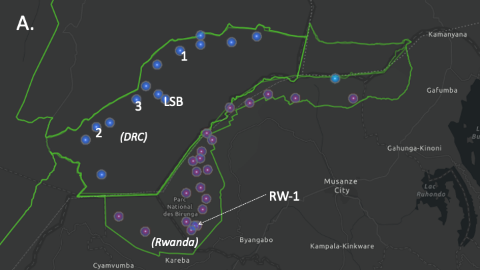

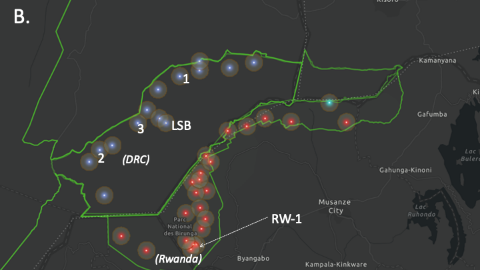

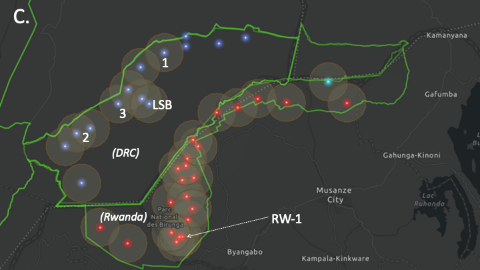


c.

b.

a.


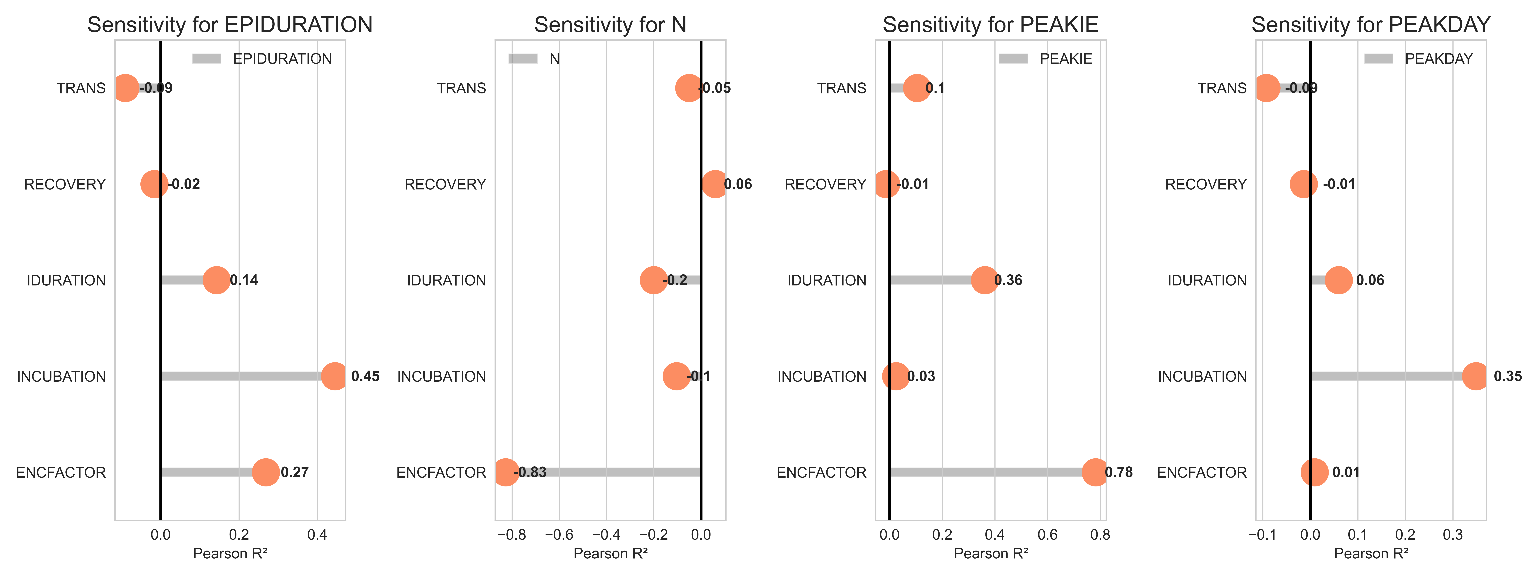


Figure S2: Sensitivity analysis results for DRC-1 scenario with encounter factor, showing Pearson's Correlation coefficient of model parameters on four model outputs.


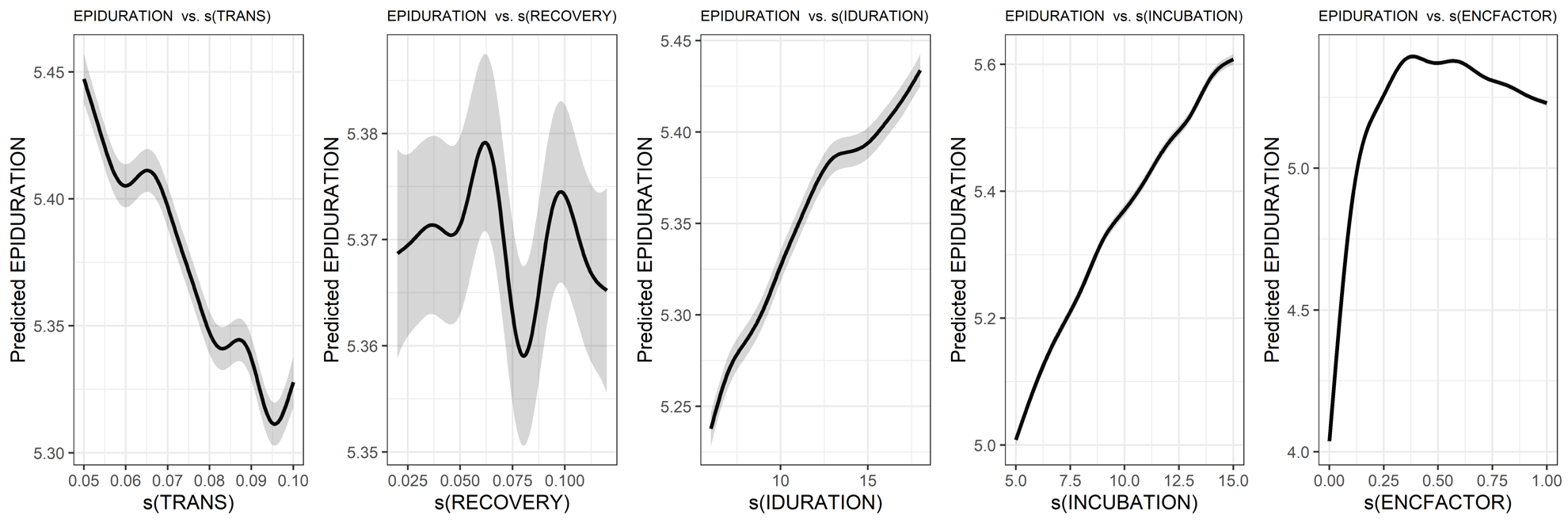


Figure S3: Sensitivity analysis results for DRC-1 scenario with encounter factor, showing GAM smooths for EPIDURATION (duration of outbreak) as outcome variable and model transmission parameters as covariates.


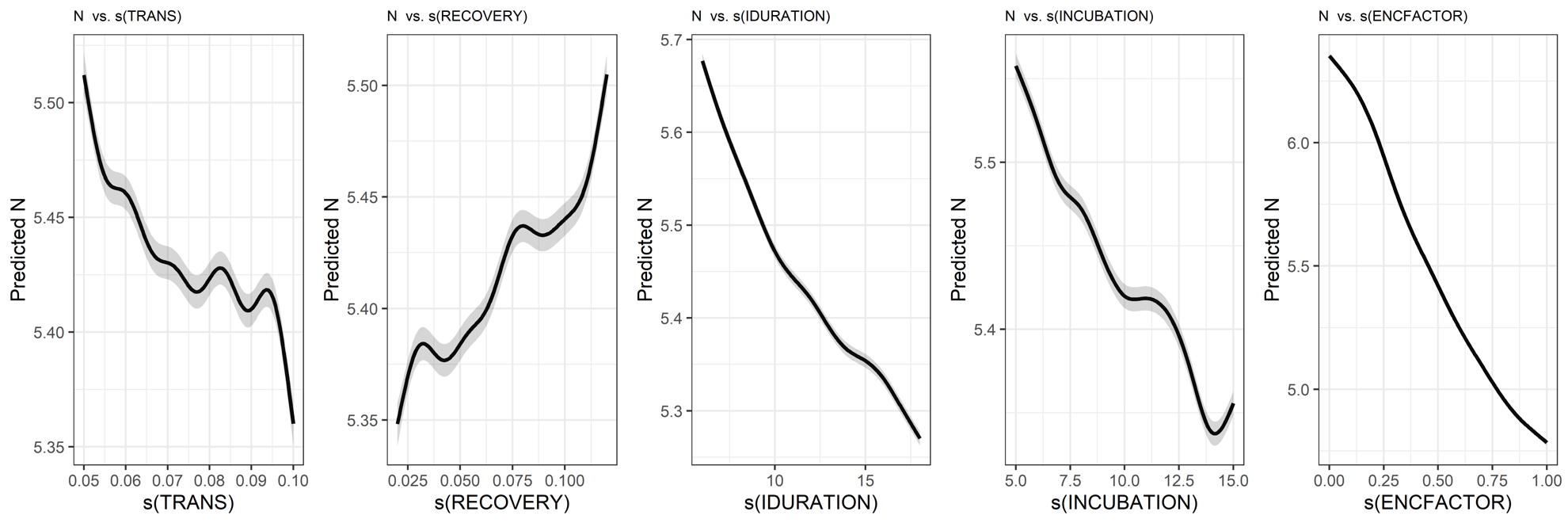


Figure S4: Sensitivity analysis results for DRC-1 scenario with encounter factor, showing GAM smooths for N (total number surviving at the end of epidemic) as outcome variable and model transmission parameters as covariates.


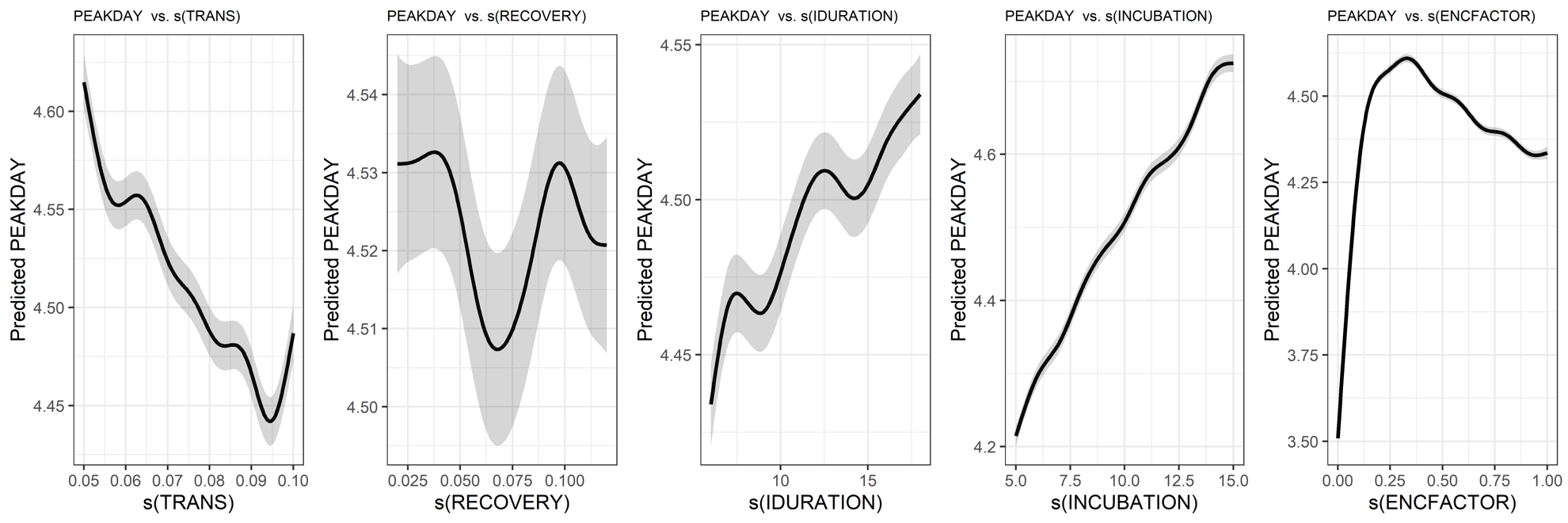


Figure S5: Sensitivity analysis results for DRC-1 scenario with encounter factor, showing GAM smooths for PEAKDAY (day with maximum number of cases) as outcome variable and model transmission parameters as covariates.


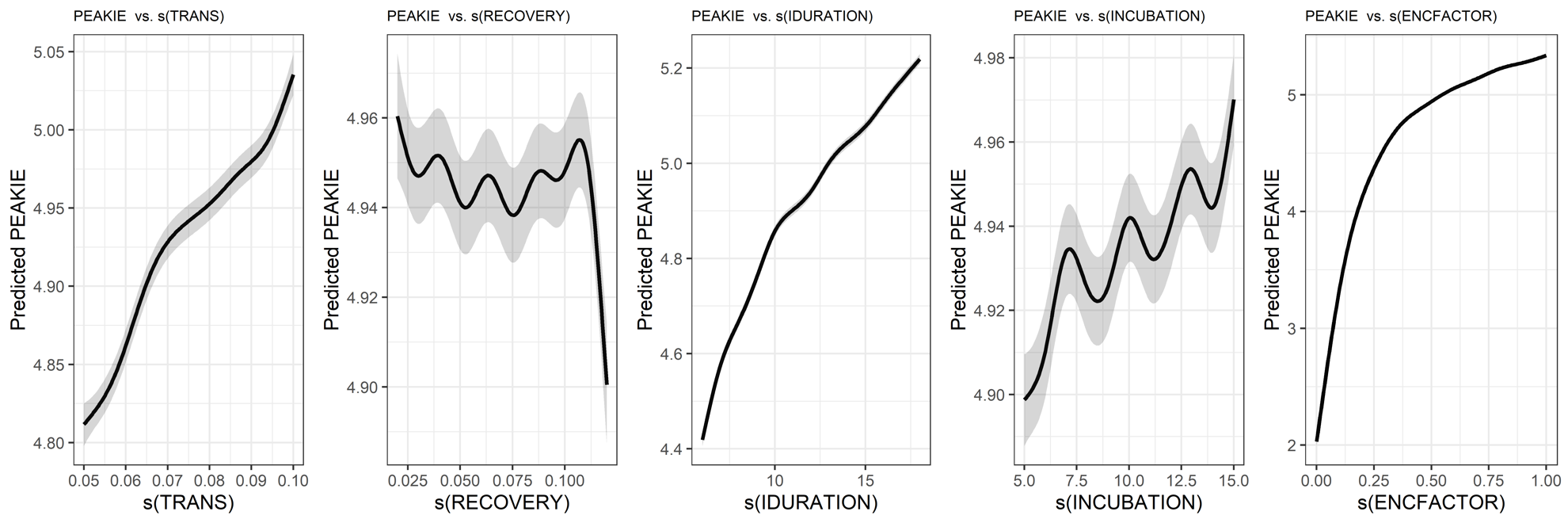


Figure S6: Sensitivity analysis results for DRC-1 scenario with encounter factor, showing GAM smooths for PEAKIE (number of infected at the peak of the epidemic) as outcome variable and model transmission parameters as covariates.


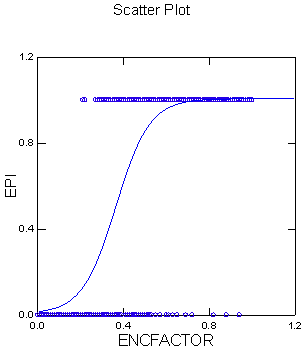


Figure S7: Logistic regression of the probability of an epidemic infecting at least 50% of the gorillas (EPI, y-axis) as a function of the encounter rate (ENCFACTOR), while keeping the core disease parameters of Transmission rate and Recovery rate within relatively narrow ranges around the baseline estimates (0.067 to 0.087 and 0.057 to 0.077, respectively). Logistic regression parameters A = -4.637, B = 12.744, r^2^ = 0.678.


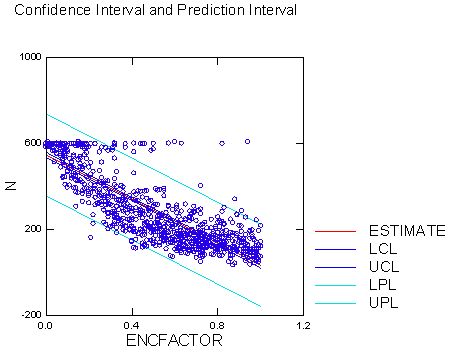


Figure S8: Linear regression the effect of the encounter rate (ENCFACTOR) on the number of gorillas surviving (N). Encounter rate would need to be less than 47% of the baseline estimate to reduce the cumulative death rate to less than 50%. Linear regression parameters A = 545.64, B = -514.02, r^2^ = 0.702. LCL and UCL = Upper and Lower Confidence Levels; LPL and UPL = Upper and Lower Prediction Levels.


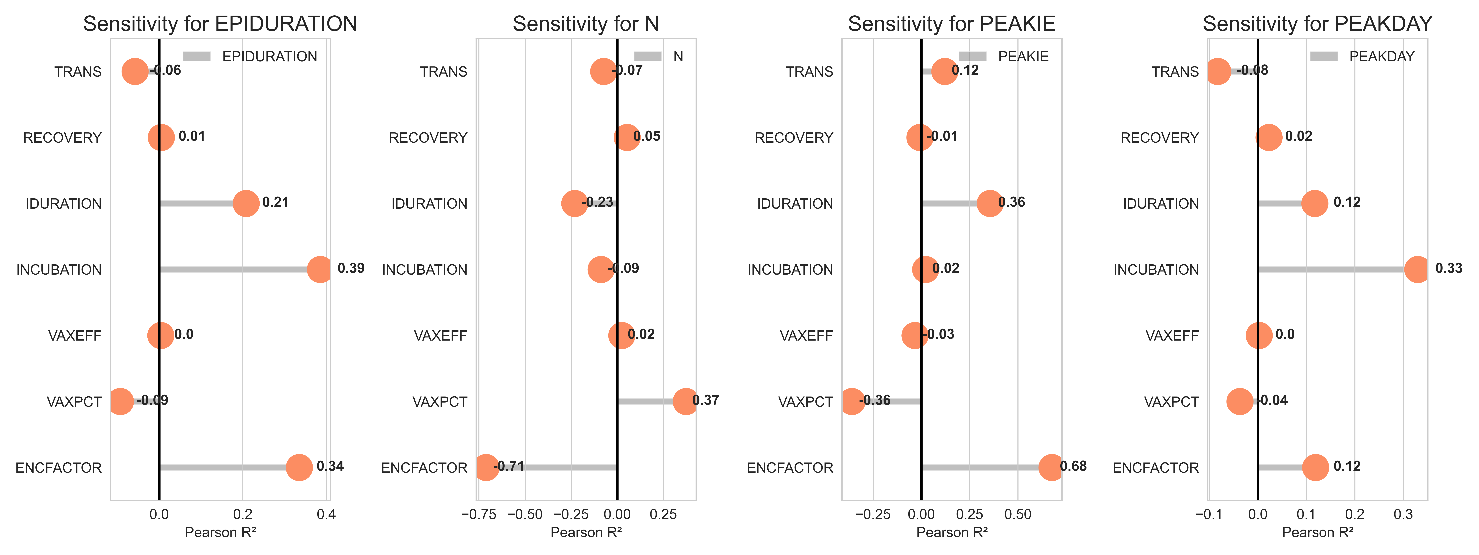


Figure S9: Sensitivity analysis results for DRC-1 vaccination scenario with encounter factor, showing Pearson's Correlation coefficient of model parameters on four model outputs.

**
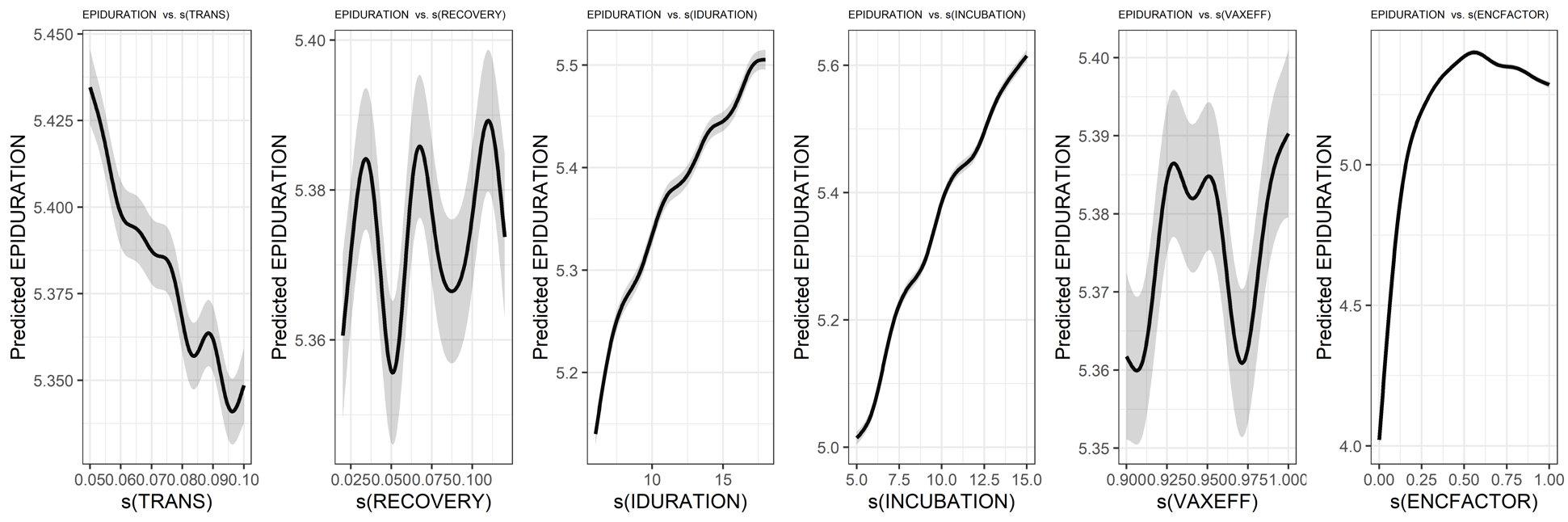
**

Figure S10: Sensitivity analysis results for DRC-1 scenario with vaccination, showing GAM smooths for EPIDURATION (duration of outbreak) as outcome variable and model transmission parameters as covariates.

**
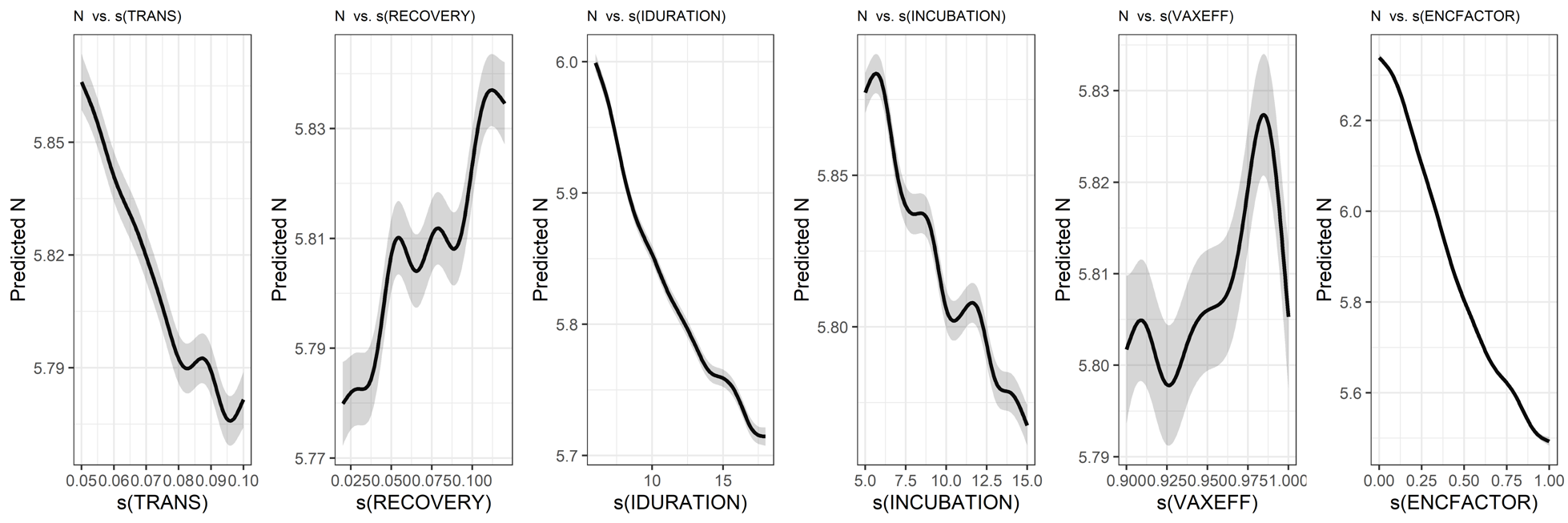
**

Figure S11: Sensitivity analysis results for DRC-1 scenario with vaccination, showing GAM smooths for N (total number survived at the end of epidemic) as outcome variable and model transmission parameters as covariates.

**
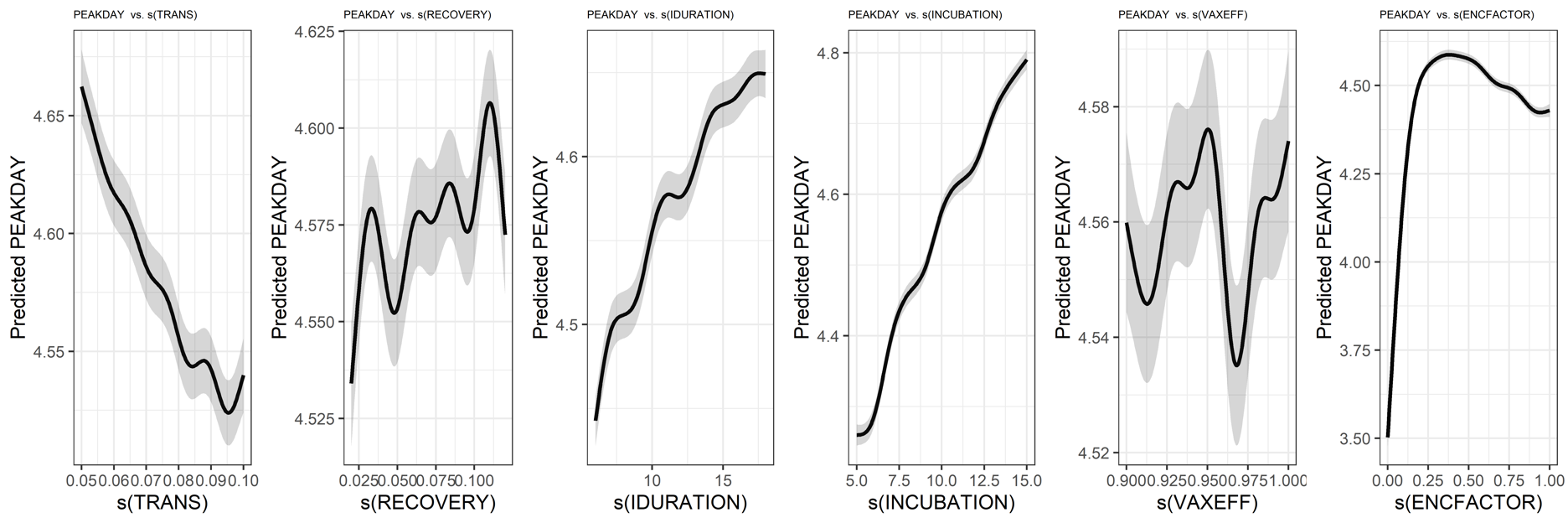
**

Figure S12: Sensitivity analysis results for DRC-1 scenario with vaccination, showing GAM smooths for PEAKDAY (day with maximum number of cases) as outcome variable and model transmission parameters as covariates.

**
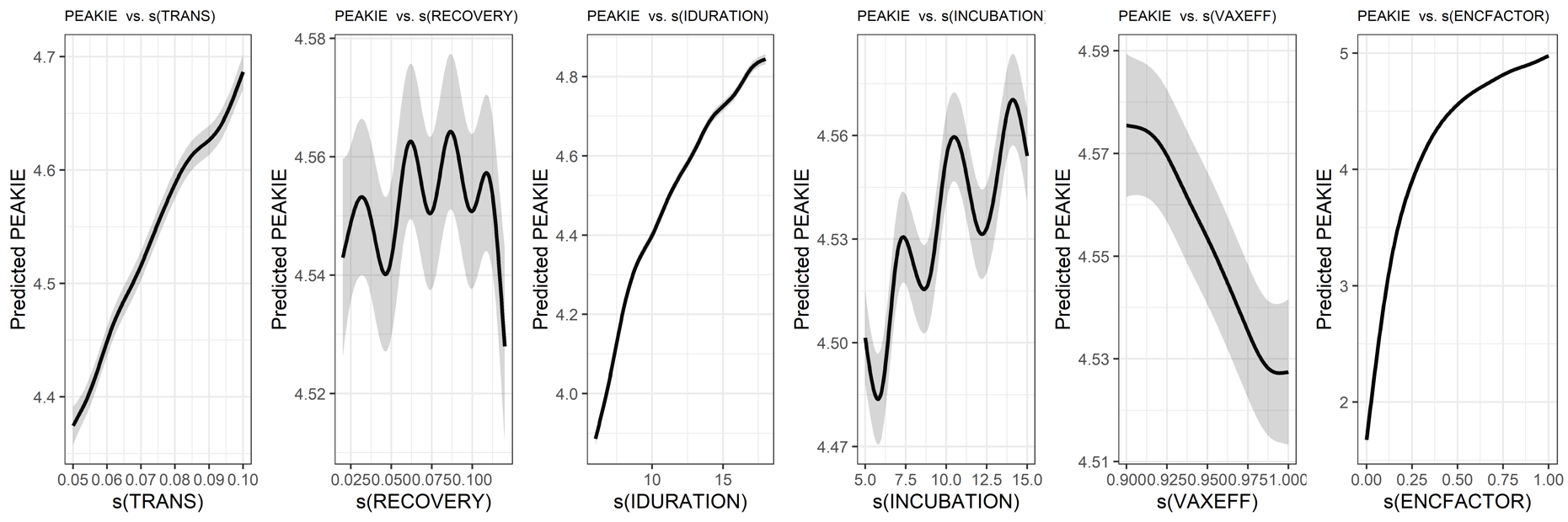
**

Figure S13: Sensitivity analysis results for DRC-1 scenario with vaccination, showing GAM smooths for PEAKIE (number of infected at the peak of the epidemic) as outcome variable and model transmission parameters as covariates.

| Table S1. Outbreak parameter values, sensitivity values tested, and results of sensitivity testing. References cited include non-human primate (NHP) and human sources depending on availability. | | | |
| --- | --- | --- | --- |
| **Parameter** | **Baseline Value** | NHP Reference | Human Reference |
| *Pre- Susceptible* (not applicable to our model as no births were included in the simulated year) | | | |
| What proportion of individuals never become susceptible | 0.00 | Unknown | EVD is almost universally fatal, with almost all fetuses miscarried, stillborn, or dying shortly after birth [1, 2] |
| Duration of pre-susceptible immunity | 0.00 | Unknown | See above Bebell *et al.* [1] and Ottoni *et al.* [2] |
| Maternal-offspring transmission | 1.00 | An Ebola-infected mother is a high-risk factor for infant chimpanzees to also be infected [3] | Young children have an increased risk of EVD if their mother is infected; this risk is higher if their mother dies, but breast feeding was not found to increase risk [4] |
| *Susceptible* | | | |
| Encounter rate as a function of distance | see below^#^ | \| Distance Between Pair of Groups \| Probability of Contact \| \| --- \| --- \| \| >1000 m \| 0 \| \| 500 – 1000 m \| 10% \| \| 250-500 m \| 25% \| \| 100 – 250 m \| 75% \| \| <100 m \| 95% \|   (E. Kambale, personal communication) | NA |
| Transmission rate given encounter | 0.067 (0.5 over 10 days) | Rizkalla *et al.* [5] estimated 50% transmission rate  1 – 0.5^(1/10)^ = .067  Caillaud *et al.* [6] estimate that the probability for a susceptible unit to get infected, per 10-day period, reached 0.22 | NA |
| *Exposed and infected* | | | |
| Incubation Period | 10 (3)^*^ | A review of Marburg and Ebola viruses in NHPs found an average incubation period of 2-14 days [7] | In humans, the range of incubation period is 2-21 days [8] with the average being 8–10 days [9] |
| *Infectious* | | | |
| Infectious period | 12 (2)^*^ | The virus has a 3-4 day carcass viability in the forest [10-11]^,^  Post-euthanasia, the virus was viable up to 7 days in experimental macaques [12] | Death 8-9 days after the onset of symptoms in patients not receiving treatment [9] |
| Probability of recovering to resistant state | 0.063 | Rizkalla *et al.* [5] averaged 3 reports of Ebola virus in great ape populations (Lossi Sanctuary gorillas, Lossi Sanctuary chimpanzees, and Minkebe gorillas) for a recovery rate of 0.067 | Although it was thought survivors were immune, there are increasing case reports of recrudescence of disease [13] |
| Probability of dying from the infection | 0.937 | Gorillas at Lokoue´ in Republic of Congo suffered 95% mortality in 2003-2004 outbreak [6]  An outbreak in Lossi (Oct 2003-Jan 2004) killed 91/95 (95.8%) newly monitored gorillas [14] | The average EVD case fatality rate is ~50% with outbreaks varying between 25% and 90% [15] |
| *Recovered / Resistant* | | | |
| Proportion acquiring permanent immunity | 1.00** | Rizkalla *et al.* [5] assumed individuals that did not die became immune | Recovered humans may be susceptible to reinfection [13] but there have been no documented cases, and antibody persistence and neutralization capacity 40 years after infection has been published [16] |
| Duration of immunity in days for those without permanent immunity | >365 | Unknown | See MacIntyre and Chughtai [13] above |
| *Vaccination* | | | |
| Efficacy | 0.97 | [Studies in progress] | Preliminary results (WHO [17]) found 97.5% vaccine efficacy in the DRC |
| Time to vaccine efficacy |  | A single dose given 3 days before challenge resulted in partial protection; if given 7 days before, complete protection was achieved [49] | In 2015, the vaccine was “100% effective” 10 days or more after administration [18] |
| Days the vaccine remains effective | >365 | [Studies in progress] | No cases following 84-day vaccine trial [19] |

^*^ Sampled from a Normal distribution with mean (SD)

^**^Given that the length of the simulation was for one year and that duration of immunity is > 365 days, immunity is functionally permanent in our model

^#^Explanation of the function used to model encounter probabilities

The function that we used in Outbreak to model the probabilities of encounters between each pair of gorilla groups was:

=IF(SIS1=IS1;0.95;

SRAND(DAY+365*(R-1)+IS1*365*500+SIS1*365*500*62)<

[(IS2=1)*(SIS2=1)*[4.702806/((DIST)^2)]

+(IS2=2)*(SIS2=2)*[0.622434/((DIST)^2)]

+(IS2!=SIS2)*[0.803210/((DIST)^2)]])

In the function syntax used by Outbreak and the variables in our model, the meaning of each of the parts of this function are as follows:

IS1 is an individual state variable that codes for the group to which a gorilla belongs;

IS1 codes for the group of the infectious individual in an encounter, and SIS1 codes for the group of the susceptible individual in an encounter;

IS2 is an individual state variable that codes for the country, with IS2 = 1 for DRC and IS2 = 2 for RW/UG;

IS2 codes for the country of the infectious individual in an encounter, and SIS2 codes for the country of the susceptible individual in an encounter;

The first part of the function shown above specifies that the encounter rate is 0.95 / day for pairs of gorillas in the same group.

The second part uses a seeded random number to generate a value between 0 and 1 that will be the same for all individuals in a pair of groups being assessed, independently generated for each day and iteration (R) of the simulation. This forces encounters between groups to affect synchronously all individuals within the groups, rather than allowing independent encounters of individuals. However, the transmission of Ebola between individuals when an encounter occurs is still determined as an independent probabilistic process, with transmission probability of 0.067.

The third part of the function specifies the probability of encounter, based on distance between mean locations, if both groups are in DRC.

The fourth part of the function specifies the probability of encounter, based on distance between mean locations, if both groups are in RW.

The last part of the function specifies the probability of encounter, based on distance between mean locations, if one group is in DRC and the other is in RW.

Distances (DIST) between mean locations are scaled to 250m units, so that they will constrain the locations to a 140 x 100 grid in the model. See main text for explanation of how the encounter-distance relationships were estimated from GPS location data. See Supplemental videos to observe examples of the pattern of spread of Ebola among the gorilla groups.

Supplemental Video 1. Video display of a sample of one iteration of the projected spread of Ebola virus over the first 180 days after it enters the Virunga Massif population of mountain gorillas via one infected individual in group DRC-1. By day 180, the disease has run its course, with recovered individuals, susceptible individuals in groups that escaped infection, and one infected individual remaining. Small jitter was added to locations of individuals, so that it could be seen when a surviving group still contained many or only one gorilla. In this iteration, six groups in RW and one lone male in DRC escaped infection. Accessible at: <https://vimeo.com/730361635>

Supplemental Video 2. Video display of a sample of one iteration of the projected spread of Ebola virus over the first 180 days after it enters the Virunga Massif population of mountain gorillas via one infected individual in group DRC-1, with 10% of the population effectively vaccinated 6 weeks after the first individual becomes sick (Infectious). By day 180, the disease has mostly run its course, with vaccinated gorillas, recovered individuals, susceptible individuals in groups that escaped infection, and a few still infectious individuals remaining. Small jitter was added to locations of individuals, so that it could be seen when a surviving group still contained many or only one gorilla. In this iteration, six groups in RW and one lone male in DRC escaped infection as of day 180, but one of the uninfected groups is close to the cluster of remaining infectious individuals. Accessible at: <https://vimeo.com/730361656>

References

1. Bebell, L. M., Oduyebo, T. & Riley, L. E. Ebola virus disease and pregnancy: A review of the current knowledge of Ebola virus pathogenesis, maternal, and neonatal outcomes. *Birth Defects Res.* **109**, 353–362 (2017).
2. Ottoni, M. P. *et al.* Ebola-negative neonates born to Ebola-infected mothers after monoclonal antibody therapy: a case series. *Lancet Child Adolesc. Health.* **4**, 884–888 (2020).
3. Formenty, P. *et al.* Ebola virus outbreak among wild chimpanzees living in a rain forest of Côte d’Ivoire. *J. Infect. Dis.* **179 Suppl 1**, S120-6 (1999).
4. Bower, H. *et al.* Effects of mother’s illness and breastfeeding on risk of ebola virus disease in a cohort of very young children. *PLoS Negl. Trop. Dis.* **10**, e0004622 (2016).
5. Rizkalla, C., Blanco-Silva, F. & Gruver, S. Modeling the impact of Ebola and bushmeat hunting on western lowland gorillas. *Ecohealth.* **4**, 151–155 (2007).
6. Caillaud, D. *et al.* Gorilla susceptibility to Ebola virus: the cost of sociality. *Curr. Biol.* **16**, R489-91 (2006).
7. Schou, S. & Hansen, A. K. Marburg and Ebola virus infections in laboratory non-human primates: a literature review. *Comp. Med.* **50**, 108–123 (2000).
8. Briand, S. *et al.* The international Ebola emergency. *N. Engl. J. Med.* **371**, 1180–1183 (2014).
9. Do, T. S. & Lee, Y. S. Modeling the spread of ebola. *Osong Public Health Res. Perspect.* **7**, 43–48 (2016).
10. Walsh, P. D., Breuer, T., Sanz, C., Morgan, D. & Doran-Sheehy, D. Potential for Ebola transmission between gorilla and chimpanzee social groups. *Am. Nat.* **169**, 684–689 (2007).
11. Leroy, E. M. *et al.* Multiple Ebola virus transmission events and rapid decline of central African wildlife. *Science.* **303**, 387–390 (2004).
12. Prescott, J. *et al.* Postmortem stability of Ebola virus. *Emerging Infect. Dis.* **21**, 856–859 (2015).
13. MacIntyre, C. R. & Chughtai, A. A. Recurrence and reinfection—a new paradigm for the management of Ebola virus disease. *Int. J. Infect. Dis.* **43**, 58–61 (2016).
14. Bermejo, M. *et al.* Ebola outbreak killed 5000 gorillas. *Science.* **314**, 1564 (2006).
15. World Health Organization. Ebola vírus disease. <https://www.who.int/en/news-room/fact-sheets/detail/ebola-virus-disease> (2021).
16. Rimoin, A. W. *et al.* Ebola Virus Neutralizing Antibodies Detectable in Survivors of theYambuku, Zaire Outbreak 40 Years after Infection. *J. Infect. Dis.* **217**, 223–231 (2018).
17. World Health Organization. Preliminary results on the efficacy of rVSV-ZEBOV-GP Ebola vaccine using the ring vaccination strategy in the control of an Ebola outbreak in the Democratic Republic of the Congo: an example of integration of research into epidemic response. https://cdn.who.int/media/docs/default-source/ebola/ebola-ring-vaccination-results-12-april-2019.pdf?sfvrsn=b9cca6aa_1&download=true (2019).
18. Woolsey, C. & Geisbert, T. W. Current state of Ebola virus vaccines: A snapshot. *PLoS Pathog.* **17**, e1010078 (2021).
19. Henao-Restrepo, A. M. *et al.* Efficacy and effectiveness of an rVSV-vectored vaccine in preventing Ebola virus disease: final results from the Guinea ring vaccination, open-label, cluster-randomised trial (Ebola Ça Suffit!). *Lancet.* **389**, 505–518 (2017).
